# 781 Eye of the Beholder: Does Visual Assessment in Severe Frostbite Accurately Predict Need for Thrombolytics?

**DOI:** 10.1093/jbcr/irad045.256

**Published:** 2023-08-29

**Authors:** Alexandra Coward, Rachel Dahl, Frederick W Endorf, Rebecca Freese, Derek Lumbard, Rachel M Nygaard, Kyle Schmitz

**Affiliations:** Hennepin Healthcare, Minneapolis, Minnesota; Hennepin County Medical Center, Minneapolis, Minnesota; Hennepin Healthcare, Minneapolis, Minnesota; University of Minnesota, Minneapolis, Minnesota; Hennepin Healthcare, Minneapolis, Minnesota; Hennepin Healthcare, Minneapolis, Minnesota; Hennepin Healthcare, Minneapolis, Minnesota

## Abstract

**Introduction:**

The most promising therapy to salvage limb length following severe frostbite injury is thrombolytics (tPA). Along with screening for contraindications, most protocols use Tc99 bone scan or angiography to identify post-rewarming perfusion deficit. However, the efficacy of tPA is time dependent and many centers lack the ability to rapidly assess perfusion in the impacted limb. Based on this, our aim was to assess perfusion using a visual assessment scale developed for the austere environment. Our aims were to assess 1) the use of this scale to identify tPA eligibility, 2) the accuracy of measured perfusion deficit using this visual scale, and 3) the predictive value of three imaging modalities to amputation level. We hypothesize that the visual scale will provide less accuracy compared to traditional perfusion assessments but could be an appropriate adjunct to assessing eligibility for tPA.

**Methods:**

Data from our prospectively maintained institutional database was used to identify a retrospective cohort of severe frostbite patients with pictures of the injury between 2016-20 (N=93). Aim 1 identified tPA eligibility based on the presence or absence of a perfusion deficit in pre-tPA visual assessment compared to pre-tPA Tc99 (N=60). Aim 2 compared the level of perfusion deficit on visual assessment to Tc99 scan (N=92). Visual assessment measure of perfusion was considered accurate if within 5% of calculated Tc99 scan. Imaging was temporally matched (i.e. pre-tPA visual to pre-tPA Tc99 and post-tPA visual to post-tPA Tc99). In aim 3 we calculated predictive values of 3 imaging modalities compared to amputation. Definitions for the predictive calculations are in figure 1.

**Results:**

Based on visual assessment alone, 17% of patients would have received tPA who did not have a post-rewarming perfusion deficit (Aim 1). When comparing the accuracy of the measure in Aim 2, only 9% of visual assessments were accurate based on Tc99 scan and a majority (67%) were over-estimations of the level of perfusion deficit. Aim 3 assessed the predictive values of imaging to amputation and are presented in Table 1.

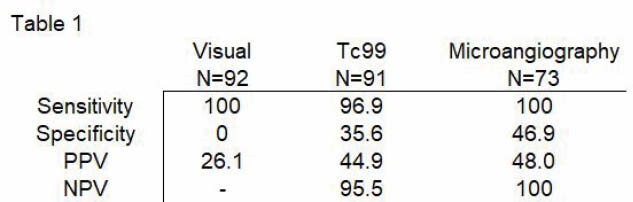

**Conclusions:**

Early visual assessment of frostbite injury lacks accuracy, even in centers with high volumes of frostbite and experienced surgeons. We found high specificity and moderate to low sensitivity with all early assessment techniques. Future studies should include methods to assess perfusion feasible for use in the low resource arena. Additional studies are needed to further elucidate the use of imaging for surgical planning following severe frostbite injury in the urban setting.

**Applicability of Research to Practice:**

Early visual assessment of severe frostbite injury lack specificity to predict amputation but may be used as an adjunct in low resource areas.

